# Evaluation of the QIAstat-Dx RP2.0 and the BioFire FilmArray RP2.1 for the Rapid Detection of Respiratory Pathogens Including SARS-CoV-2

**DOI:** 10.3389/fmicb.2022.854209

**Published:** 2022-03-24

**Authors:** Hayley Cassidy, Mart van Genne, Erley Lizarazo-Forero, Hubert G. M. Niesters, Lilli Gard

**Affiliations:** Division of Clinical Virology, Department of Medical Microbiology and Infection Prevention, University Medical Center Groningen, University of Groningen, Groningen, Netherlands

**Keywords:** QIAstat-Dx, BioFire FilmArray, molecular diagnostics, respiratory infections, syndromic testing, point-of-care (POC), SARS-CoV-2

## Abstract

Point-of-care syndromic panels allow for simultaneous and rapid detection of respiratory pathogens from nasopharyngeal swabs. The clinical performance of the QIAstat-Dx Respiratory SARS-CoV-2 panel RP2.0 (QIAstat-Dx RP2.0) and the BioFire FilmArray Respiratory panel RP2.1 (BioFire RP2.1) was evaluated for the detection of SARS-CoV-2 and other common respiratory pathogens. A total of 137 patient samples were retrospectively selected based on emergency department admission, along with 33 SARS-CoV-2 positive samples tested using a WHO laboratory developed test. The limit of detection for SARS-CoV-2 was initially evaluated for both platforms. The QIAstat-Dx RP2.0 detected SARS-CoV-2 at 500 copies/mL and had a positive percent agreement (PPA) of 85%. The BioFire RP2.1 detected SARS-CoV-2 at 50 copies/mL and had a PPA of 97%. Both platforms showed a negative percent agreement of 100% for SARS-CoV-2. Evaluation of analytical specificity from a range of common respiratory targets showed a similar performance between each platform. The QIAstat-Dx RP2.0 had an overall PPA of 82% (67–100%) in clinical samples, with differences in sensitivity depending on the respiratory target. Both platforms can be used to detect acute cases of SARS-CoV-2. While the QIAstat-Dx RP2.0 is suitable for detecting respiratory viruses within a clinical range, it has less analytical and clinical sensitivity for SARS-CoV-2 compared to the BioFire RP2.1.

## Introduction

Respiratory infections are a significant source of morbidity and mortality in hospitals and in the community, accounting for numerous hospital admissions every year (Beninca et al., 2017; Rijn et al., 2018). A major challenge is that respiratory infections, regardless of pathogen, often present with similar symptoms rendering an initial diagnosis difficult (Popowitch et al., 2013). A timely diagnosis is crucial for patient management, optimizing length of hospital admission, and preventing transmission. This is particularly important for the severe acute respiratory syndrome coronavirus 2 (SARS-CoV-2) pandemic. In addition, a shorter length of stay (LOS) can reduce incidences of nosocomial infections and unnecessary antibiotic use (Dik et al., 2016; Wolkewitz et al., 2019).

The need for rapid syndromic testing has led to the rise of multiplex real-time PCR systems which are highly automated and perform all steps in a self-contained device. Systems such as the BioFire FilmArray (BioMérieux, Marcy-l’Étoile, France), Xpert Xpress (Cepheid, Sunnyvale, CA, United States), ePlex (GenMark Diagnostics, Roche, Basel, Switzerland), VERIGENE (Lumine DiaSorin, Saluggia, Italy), and the QIAstat-Dx (Qiagen, Hilden, Germany) are frequently used for patients exhibiting more than one respiratory symptom (Popowitch et al., 2013; Pinsky and Hayden, 2019). Screening for multiple pathogens simultaneously not only contributes to improving diagnostic stewardship and minimizing antibiotic use, but also enables seasonal tracking and identification of less common respiratory pathogens (Meyers et al., 2020).

Prior to the SARS-CoV-2 pandemic, the University Medical Center Groningen (UMCG) had incorporated the BioFire Respiratory Panel (RP) RP2.0 into routine diagnostics. Integration of point-of-care (POC) tests like the BioFire aid the “€hr” concept, where cost per sample is multiplied by the total turnaround time (Poelman et al., 2020). However, amplification data such as cycle threshold (Ct) values are not reported in the automatic output results (Poritz et al., 2011). Therefore, semi quantitative and qualitative information which may be useful for patient management is missing.

The QIAstat-Dx Respiratory SARS-CoV-2 panel RP2.0 (QIAstat-Dx RP2.0) (Qiagen, Germany) and the BioFire FilmArray Respiratory panel RP2.1 (BioFire RP2.1) have recently emerged in the market, generating results from 22 different respiratory targets, including SARS-CoV-2 in approximately 1 h (Rao et al., 2021). While the BioFire RP2.1 offers relatively quicker results (45 vs. 70 min), the QIAstat-Dx RP2.0 also offers direct introduction of the nasopharyngeal swab into the cartridge, reducing hands-on-time. Furthermore, the QIAstat-Dx RP2.0 also offers cycle threshold (Ct) values as an automatic output result, providing additional qualitative information and permitting further comparison with the laboratory developed test (LDT) (Boers et al., 2021). As the QIAstat-Dx and the BioFire are prominent platforms in the market, it is important to understand their strengths and weakness to ensure the quality of data collected. Continually evaluating and implementing new products for routine testing is crucial for diagnostics to move forward. The aim of the study was to compare the QIAstat-Dx RP2.0 with the BioFire RP2.1 in relation to analytical specificity, analytical sensitivity and clinical sensitivity for the detection of respiratory pathogens.

## Materials and Methods

### Distribution of SARS-CoV-2 Positive Samples at the University Medical Center Groningen

To establish an overall baseline SARS-CoV-2 viral load in the samples, the distribution of Ct values [which are highly associated with viral load (Walker et al., 2021)] from the SARS-CoV-2 RT-qPCR were retrospectively collected from January 2020 to May 2021. A study list including Ct values from three different populations was generated using the laboratory information management system (GLIMS): patients, hospital workers, and individuals utilizing public health services. All three populations offered different clinical backgrounds and could benefit from rapid testing.

### Patient Inclusion for Syndromic Testing

A retrospective study was conducted in patients admitted to the UMCG during the 2019/2020 respiratory season from the patient database system (EPIC) (Supplementary Tables 1, 2). Patients who presented with two or more respiratory symptoms, including a cough, cold, sore throat, fever, or a temperature above 38°C were included. A total of 137 patient nasopharyngeal swabs (NPS) previously tested through the BioFire RP2.0 (not including the SARS-CoV-2 target) (BioMérieux), were retrospectively selected based on emergency department admission and co-detections (two or more respiratory targets detected). Rapid testing is crucial in emergency departments, coupled with the fact that this hospital is a major transplant center and caters for patients likely to have a higher number of infections/co-infections (Fischer, 2016). In total, single respiratory infections (*n* = 104), multiple respiratory infections (co-infections) (*n* = 16) and negative respiratory samples (*n* = 17) were included. All negative specimens were additionally tested on the SARS-CoV-2 LDT to confirm specimens tested negative on all available platforms. Patients ranged from 0 to 97 years of age. Additionally, 33 SARS-CoV-2 positive samples from patients (33–88 years) with a symptomatic SARS-CoV-2 infection, as determined by the patient database system (EPIC), were tested through the LDT between March and May 2020 and were included in the study. All samples included were anonymized and aliquoted to ensure no differences in the number of freeze-thaw cycles. Discrepant results were repeated if there was sufficient sample volume available. The local UMCG Ethics Committee approved this non-WMO study under the waiver “METc-2009.169.”

### Laboratory Developed Test (SARS-CoV-2)

Real-time reverse transcriptase PCR (RT-qPCR) testing was performed on NPS by targeting the envelope gene (E-gene) of SARS-CoV-2 using a reference assay from the World Health Organization (Corman et al., 2020). Briefly, nucleic acids were extracted from 190 μL of sample using the NucliSense EasyMag (BioMérieux) and eluted in 110 μL. A total of 10 μL of phocine distemper virus (PDV), which severed as an internal control (IC), was added prior to isolation (Poelman et al., 2015). The TaqMan Fast Virus 1-Step kit (ThermoFisher Scientific, Waltham, MA, United States) was used along with 10 μL of extracted RNA to create a total reaction volume of 25 μL. The following PCR cycling conditions were performed on an ABI 7500 (Life Technologies, Carlsbad, CA, United States): 15 min at 50°C, 20 s at 95°C, followed by 45 cycles of 5 s at 95°C, 5 s at 50°C, and 45 s at 60°C. Analysis of LDTs was completed using the 7500 System SDS Software (v1.4).

### Laboratory Developed Respiratory Screen

A RT-qPCR respiratory screen previously implemented at the UMCG is routinely used to screen and provide Ct values for 16 respiratory targets: Influenza A virus, Influenza B virus, parainfluenza virus 1-4, human rhinovirus/enterovirus, coronaviruses OC43, NL63, 229E, HKU1, respiratory syncytial virus A and B, adenovirus, bocavirus, and human metapneumovirus. Briefly, nucleic acids were extracted from 190 μL of sample using the NucliSense EasyMag (BioMérieux) and eluted in 110 μL. PDV severed as an IC. A multiplex RT-qPCR using the TaqMan Fast Virus 1-Step kit (ThermoFisher Scientific) was performed using 10 μL of each extracted viral RNA in a total reaction volume of 25 μL (Supplementary Table 3). Amplification was performed on an ABI7500 (Life Technologies) with the following PCR cycling conditions: 2 min 50°C, 20 s 95°C, followed by 45 cycles of 3 s 95°C and 32 s at 60°C. To differentiate between a rhinovirus and enterovirus following the BioFire RP2.0 or respiratory screen, a RT-qPCR targeting the 5’NTR region was performed (see Supplementary Methods).

### QIAstat-Dx RP2.0

Retrospective testing of the 170 NPS was performed using the QIAstat-Dx, according to the manufacturer’s instructions (Qiagen, Hilden, Germany). Briefly, 300 μL of the sample was transferred into the main port on the cartridge which was then loaded into the QIAstat-Dx. Amplification signals were analyzed with the QIAstat-Dx Analyzer v1.3.0. Results for each of the 19 viral and 3 bacterial targets (Supplementary Table 4) were generated within 70 min. In addition to including an IC, the QIAstat-Dx also generates Ct values as an additional qualitative measurement (Visseaux et al., 2020). The QIAstat-Dx panel targets two genes in SARS-CoV-2, the Orfb gene and the E gene, which are detected with the same fluorescence channel.

### BioFire RP2.1

Retrospective testing of the 50 NPS (33 SARS-CoV-2 and 17 clinically negative samples) was performed using the BioFire, according to the manufacturer’s instructions (BioMérieux). Briefly, 300 μL of the sample was added to 3 mL of viral transport medium and loaded into the BioFire pouches. Results for each of the 18 viral and 4 bacterial targets (Supplementary Table 4) were generated within 45 min. For quality control, the BioFire has its own IC. The BioFire has three independent assays to detect SARS-CoV-2: two regions in the Orf1ab gene and one region in the ORF8 gene.

### Evaluating Sensitivity

The analytical sensitivity of a diagnostic assay typically describes the lowest point at which the pathogen of interest can still be accurately measured (Vandenberg et al., 2021). To evaluate the analytical sensitivity of the QIAstat-Dx RP2.0 and the BioFire RP2.1 for SARS-CoV-2, the Analytical Quality Control for Molecular Diagnostics Panel 01 (SCV2AQP01-A), containing digital PCR data (in digital copies/mL [dC/mL]) (Qnostics, Glasgow, United Kingdom) was used and compared with the SARS-CoV-2 LDT.

The clinical sensitivity of a diagnostic assay additionally takes into account factors such as sample collection and composition of patient material. To evaluate the SARS-CoV-2 clinical sensitivity of the QIAstat-Dx RP2.0 and BioFire RP2.1, patient samples with a positive LDT detection were selected from a range of Ct values (Ct 13–32) and tested in duplicate. Clinical sensitivity of the QIAstat-Dx RP2.0 for single (*n* = 104) and multi-infections (*n* = 16), other than SARS-CoV-2, from patient samples was additionally evaluated. These infections had been initially confirmed through the BioFire RP2.0. Only the QIAstat-Dx RP2.0 was investigated as it had not been previously implemented into diagnostics. Clinical sensitivity and the limit of detection (LOD) of the QIAstat-Dx RP2.0 was additionally explored for dual infections by creating artificial co-infections from common respiratory pathogens (see Supplementary Material).

### Evaluating Specificity

The analytical specificity of a diagnostic assay measures the presence of off-target pathogens (Vandenberg et al., 2021). To evaluate the analytical specificity and cross-reactivity, two Q control panels (RTX1-5QC01-A and B) from Qnostics containing a combination of common (and genetically similar) respiratory pathogens were tested using the QIAstat-Dx RP2.0, BioFire RP2.1, and LDT respiratory panel as a gold standard. To evaluate the clinical specificity, patient samples (*n* = 17) previously found negative on the BioFire RP2.0 and the SARS-CoV-2 LDT, were also evaluated in the QIAstat-Dx RP2.0 and the BioFire RP2.1.

## Results

### SARS-CoV-2 Positive Samples at the University Medical Center Groningen

**FIGURE 1:**
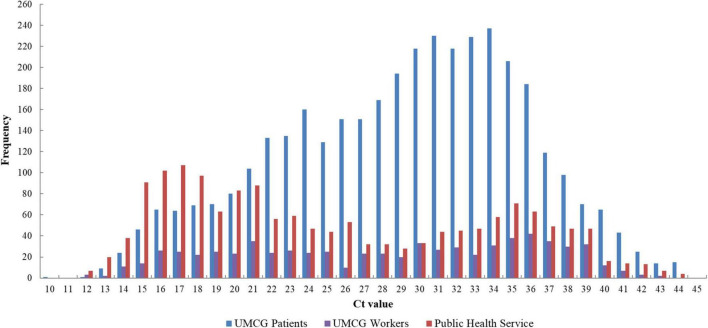
Distribution of Ct values of SARS-CoV-2 positive patients from January 2020 to May 2021 (*n* = 6,035). RT-qPCR was performed by targeting the E gene of SARS-CoV-2. UMCG patient Ct values also contain follow-up samples (in-patients and out-patients). The Ct values plotted included both symptomatic and non-symptomatic detections. UMCG, University Medical Center Groningen; Ct, cycle threshold.

Between January 2019 and May 2021, a total of 86,076 samples were tested for SARS-CoV-2 using the LDT (UMCG patients, UMCG workers or Public Health Service users). A total of 6,035 (7%) samples had a Ct range from Ct 10 to 44 (Figure 1). Although SARS-CoV-2 Ct values were normally distributed, UMCG patients tended to have higher Ct values, with an average Ct value of 29 and a highest frequency at Ct value 34. SARS-CoV-2 Ct values from UMCG workers remained constant with few peaks (average Ct value of 28). Meanwhile, SARS-CoV-2 Ct values from the Public Health Service tended to be lower, with an average Ct value of 25.

### Evaluating SARS-CoV-2 on the QIAstat-Dx RP2.0 and the BioFire RP2.1

#### Analytical Sensitivity of SARS-CoV-2

The analytical performance of the QIAstat-Dx RP2.0 and the BioFire RP2.1 was evaluated using the Analytical Q panel for SARS-CoV-2 (Table 1). The QIAstat-Dx RP2.0 was able to detect SARS-CoV-2 at 1000 dC/mL (Ct 30.6) in duplicate and 500 dC/mL (Ct 31.9) singly (Table 1). Meanwhile, the BioFire RP2.1 could detect SARS-CoV-2 at 50 dC/mL (Ct 35) in duplicate and was comparable to the LDT (Table 1). Both platforms did not detect SARS-CoV-2 in the negative control.

**TABLE 1:** Analytical sensitivity of the QIAstat-Dx RP2.0 and BioFire RP2.1 for SARS-CoV-2.

QCMD analytical panel	dC/mL	LDT (Ct value)	QIAstat-Dx RP2.0 (Ct value)	BioFire RP2.1
SCVA2AQP01-S01	1000000	21.9	29.9/29.6	Detected
SCVA2AQP01-S02	100000	24.7	30.5/34.2	Detected
SCVA2AQP01-S03	10000	27.9	32.5/35.7	Detected
SCVA2AQP01-S04	5000	28.5	35.5/36.7	Detected
SCVA2AQP01-S05	1000	30.6	36.2/35.7	Detected
SCVA2AQP01-S06	500	31.9	33.5/ND	Detected
SCVA2AQP01-S07	100	34.3	ND	Detected
SCVA2AQP01-S08	50	35.0	ND	Detected
SCVA2AQP01-S09*^1^	–	ND	ND	ND

QCMD, Quality Control for Molecular Diagnostics; dC, digital copies; LDT, laboratory developed test; Ct, Cycle threshold; ND, not detected. *^1^Negative control.

#### Clinical Sensitivity and Specificity of SARS-CoV-2

**FIGURE 2:**
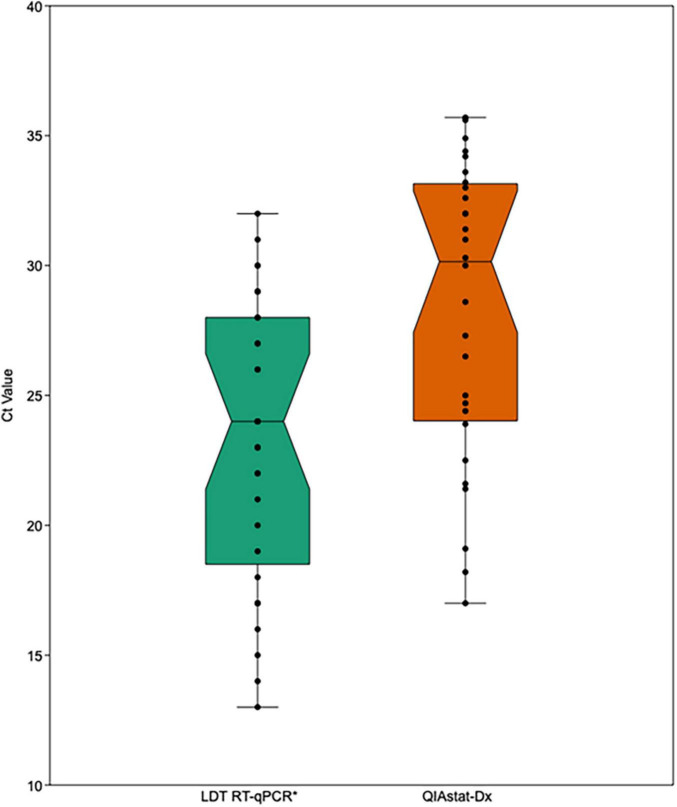
Comparison of SARS-CoV-2 Ct values from the LDT RT-qPCR and the QIAstat-Dx (RP2.0). The box represents the upper and lower quartiles, the horizontal line represents the median and the standard error is shown by upper and lower whisker lines. LDT, laboratory developed test; Ct, cycle threshold. *Gold-standard.

The performance of the QIAstat-Dx RP2.0 and BioFire RP2.1 were evaluated using SARS-CoV-2 positive samples (*n* = 33) and clinically negative samples (*n* = 17), previously tested through the LDT. Overall, the sensitivity for the QIAstat-Dx RP2.0 and the BioFire RP2.1 was 85 and 97%, respectively. Four samples tested on the QIAstat-Dx RP2.0 (Ct 22, 29, 29 and 32) and one sample tested on the BioFire RP2.1 (Ct 23) was found negative, despite repeat testing. Additionally, two samples tested in the QIAstat-Dx RP2.0 had system errors. Only one sample with a system error could be repeated and was subsequently found positive for SARS-CoV-2. Meanwhile, the overall specificity for the QIAstat-Dx RP2.0 and the BioFire RP2.1 was 100%. The Ct values for the QIAstat-Dx RP2.0 were subsequently higher than those reported by the LDT (28.25 ± 5.75 vs. 23.27 ± 5.33) (Figure 2). The IC in the QIAstat-Dx RP2.0 ranged from Ct 32 to 36.5.

#### Analytical Specificity

The analytical specificity of the QIAstat-Dx RP2.0 and BioFire RP2.1 could be evaluated by assessing two Q control multiplex panels containing combinations of common respiratory pathogens, including closely related viruses (Tables 2, 3).

**TABLE 2:** Qnostics respiratory panel 1.

Pool	Targets in mix	LDT	QIAstat-Dx RP2.0	BioFire RP2.1
		Ct value	Ct value	Detected
1	Parainfluenza virus 1	16.6	21.5	Detected
	Parainfluenza virus 2	25.0	ND	Detected
	Parainfluenza virus 3	26.0	ND	Detected
	Parainfluenza virus 4	28.7	ND	Detected
2	Influenza A virus H1N1	31.4	32.0	Detected
	Influenza B virus (Victoria)	31.4	35.6	Detected
	Respiratory syncytial virus A	32.1	31.6	Detected
	SARS-CoV-2	26.5	33.5	Detected
3	Coronavirus OC43	27.0	28.6	Detected
	Coronavirus 229E	24.8	28.8	Detected
	Coronavirus NL63	24.6	28.9	Detected
	Coronavirus HKU1	26.4	32.3	Detected
4	Rhinovirus 5	21.2	31.2	Detected
	Enterovirus D68	25.3	31.2	Detected
	Adenovirus 1	30.3	34.9	Detected
5	*Mycoplasma pneumoniae*	N/A	28.3	Detected
	Legionella pneumoniae*	N/A	30.8	N/A
	*Bordetella pertussis*	N/A	29.3	Detected

LDT, laboratory developed test (respiratory screen); Ct, cycle threshold; ND, not detected; N/A, not applicable. *The Legionella pneumonia target is not currently present in BioFire RP2.1. Pool 5 could not be directly compared as the bacterial targets are not present in the LDT respiratory screen.

**TABLE 3:** Qnostics respiratory panel 2.

Pool	Targets in mix	LDT	QIAstat-Dx RP2.0	BioFire RP2.1
		Ct value	Ct value	Detected
1	Influenza A virus H1N1	32.0	37.0	Detected
	Influenza B virus (Victoria)	32.0	34.8	Detected
	Respiratory syncytial virus A	32.1	33.6	Detected
	SARS-CoV-2	26.5	34.9	Detected
2	Parainfluenza virus 1	25.8	28.7	Detected
	Adenovirus 1	ND	32.1	Detected
	*Mycoplasma pneumoniae*	N/A	31.7	Detected
	Coronavirus OC43	29.4	33.2	Detected
3	Parainfluenza virus 2	24.9	28.7	Detected
	Metapneumovirus A2	27.5	30.4	Detected
	Enterovirus A16	23.1	30.9	Detected
	Coronavirus 229E	28.1	36.6	Detected
4	Parainfluenza virus 3	26.9	27.5	Detected
	Rhinovirus 16	26.9	32.7	Detected
	*Legionella pneumoniae*	N/A	30.4	N/A
	Coronavirus NL63	28.4	31.9	Detected
5	Parainfluenza virus 4	26.6	30.7	Detected
	Adenovirus 14	33.8	34.8	Detected
	Respiratory syncytial virus B	24.5	29.4	Detected
	Enterovirus D68	24.9	31.9	Detected

*LDT, laboratory developed test (respiratory screen); Ct, cycle threshold; ND, not detected; N/A, not applicable.*

Only parainfluenza virus 1 was detected by the QIAstat-Dx RP2.0 in pool 1, suggesting a potential problem in competition between the parainfluenza virus targets (Table 2). Repeat testing of pool 1 on the QIAstat-Dx RP2.0 yielded a similar result. All other viral mixes were detected by the QIAstat-Dx RP2.0 and the BioFire RP2.1. The QIAstat-Dx RP2.0, on average, reported 4.5 Ct values higher (−0.54 to 9.99), compared to the LDT.

All virus targets were detected by the QIAstat-Dx RP2.0 and the BioFire RP2.0 in panel 2 (Table 3). Similar to panel 1, Ct values on the QIAstat-Dx RP2.0 were on average 4.4 Ct values higher (0.65–8.49), compared to the LDT. Parainfluenza viruses 1–4 were detected individually in panel 2, which suggests a problem with primer specificity within these targets.

### Evaluating Patient Respiratory Infections on the QIAstat-Dx RP2.0

#### Single Respiratory Infections on the QIAstat-Dx RP2.0

The performance of the QIAstat-Dx RP2.0 individual targets were evaluated in clinical samples previously run on the BioFire RP2.0 and contained single respiratory detections (*n* = 137) (Table 4).

**TABLE 4:** Performance of the QIAstat-Dx RP2.0 on single infections.

Target	Number of samples	Positive results on the QIAstat-Dx RP2.0	PPA (%)
Adenovirus	1	1	100
Coronavirus 229E	1	1	100
Coronavirus HKU1	3	3	100
Coronavirus NL63	6	4	67
Coronavirus OC43	5	5	100
Human metapneumovirus	9	9	100
Human rhinovirus/enterovirus	27	18*^1,^*^2^	67
Influenza A virus H1N1	8	6	75
Influenza A virus H3	16	15	94
Influenza A virus	2	2	100
*Mycoplasma pneumonia*	3	3	100
Parainfluenza virus 1	8*^3^	6	75
Parainfluenza virus 2	1	1	100
Parainfluenza virus 4	3	3	100
Respiratory syncytial virus A	11	9	82
SARS-CoV-2	33	27*^4,^*^5^	82
Total	137	113	82

*^1^Internal control fail (n = 1), *^2^Miss-match (n = 1) (should have been coronavirus NL63), *^3^Fail (n = 1), *^4^internal control failure (n = 1), *^5^Cartridge failure (n = 1). PPA, positive percent agreement.

Overall a PPA of 82% (67–100%) was achieved for the QIAstat-Dx RP2.0, when compared to the BioFire RP2.0 (Table 4). Maximum concordance with the BioFire RP2.0 was achieved in 9/16 respiratory targets: adenovirus (1/1), coronavirus 229E (1/1), coronavirus HKU1 (3/3), coronavirus OC43 (5/5), human metapneumovirus (9/9), influenza A virus (1/1), *Mycoplasma pneumoniae* (1/1), parainfluenza virus 2 (1/1), and parainfluenza virus 3 (3/3). However, some targets had a lower sensitivity (PPA < 80%). This was observed for human rhinovirus/enterovirus (18/27), coronavirus NL63 (4/6), influenza A virus H1N1 (6/8), and parainfluenza virus 1 (6/8).

#### Multiple Respiratory Infections on the QIAstat-Dx RP2.0

Patient samples were selected based on a co-infection result on the BioFire RP2.0 and tested on the QIAstat-Dx RP2.0 (*n* = 16) (Table 5). In cases of discrepancy from the BioFire RP2.0, samples with enough volume were repeated on the QIAstat-Dx RP2.0 and LDT (respiratory screen).

**TABLE 5:** Performance of the QIAstat-Dx RP2.0 on multiple infections.

Sample no.	Targets previously detected on the BioFire RP2.0	Targets detected on the QIAstat-Dx RP2.0	QIAstat-Dx RP2.0 match to BioFire RP2.0		Targets detected on the LDT following discrepancy (Ct value)
			Run 1	Run 2	
1	HRV/EV and PIV1	PIV1	No	N/A	HRV (31), PIV1 (21)
2	HRV/EV and PIV4	PIV4	No	N/A	HRV (28), PIV4 (22)
3	HRV/EV and RSV	HRV/EV and RSV	Yes	N/A	HRV (28), RSV (26)
4	HRV/EV and INFH3	INFAH3	No	No	HRV (43), INFH3 (23)
5	HRV/EV and PIV4	HRV/EV	No	No	HRV (22), PIV4 (38)
6	HRV/EV and CoV-OC43	HRV/EV	No	Yes	HRV (33), CoV-OC43 (32)
7	HRV/EV, AV, and RSV	RSV	No	Yes	HRV (neg), RSV (17)
8	HRV/EV and RSV	Fail	No	N/A	HRV (22), RSV (17)
9	HRV/EV and INFH3	HRV/EV and INFH3	Yes	N/A	HRV (34), INFH3 (18)
10	HRV/EV and BP	HRV/EV and BP	Yes	N/A	HRV (27)
11	HRV/EV, AV, and INFH3	INFH3	No	N/A	EV (34), INFH3 (17)
12	HRV/EV and INFH3	INFH3	No	N/A	N/A
13	CoV-OC43 and RSV	CoV-OC43	No	N/A	CoV-OC43 (32), RSV (neg)
14	CoV-OC43, INFH3, CoV-HKU1	INFH3 and CoV-HKU1	No	No	CoV-OC43 (neg), INFH3 (18), CoV-HKU1 (28)
15	CoV-NL63 and INFAH3	CoV-NL63 and INFAH3	Yes	N/A	CoV-NL63 (neg), INFH3 (28)
16	CoV-NL63 and INFAH3	CoV-NL63 and INFAH3	Yes	N/A	CoV-NL63 (29), INFH3 (21)

*HRV/EV, Human rhinovirus/enterovirus; AV, Adenovirus; BP, Bordetella pertussis; PIV1, Human Parainfluenza Virus Type 1; PIV4, Human Parainfluenza Virus Type 4; RSV, Respiratory syncytial virus; INFH3, Influenza A subtype H3; CoV-OC43, coronavirus OC43; AV, adenovirus; HKU1, Coronavirus HKU1; CoV-NL63, coronavirus NL63; Ct, cycle threshold; N/A, not available (not enough sample volume). Adenovirus and Bordetella pertussis targets are not present on the LDT respiratory screen.*

The QIAstat-Dx RP2.0 had a 44% concordance with the BioFire RP2.0, with all multiple infections detected in 7/16 patient samples. However, in four patient samples (7, 13, 14, and 15), repeat testing using the LDT yielded a negative result for one of the targets identified with the BioFire (RP2.0). For two of these samples (13 and 14), the QIAstat-Dx RP2.0 yielded a similar result to the LDT. A co-infection with HRV/EV occurred most frequently within this dataset with 75% (*n* = 12) of patient samples. A further LDT revealed only sample 11 had an enterovirus, while the remaining samples had a rhinovirus. As shown in Table 5, discordant results were found in combinations of HRV/EV with: PIV1, PIV4, INFH3, RSV, and AV. In each of these cases, the QIAstat-Dx RP2.0 failed to detect the HRV/EV target. These results indicate a potential problem with sensitivity or internal competition in samples with multiple respiratory detections. As a result of this finding, clinical sensitivity of dual infections was further evaluated by creating three artificial panels, each containing two common respiratory targets detected from patient’s samples (Supplementary Figure 1). The majority of viral targets had a LOD of Ct 37 (Ct 35.4–38.1) on the QIAstat-Dx RP2.0. However, detection was not always linear with the dilution factor fluctuating depending on the viral target, which could lead to discrepant results and challenges in detecting samples with higher Ct values (Supplementary Figure 1).

## Discussion

Point-of-care testing is becoming more readily implemented in routine diagnostics, with infectious etiologies simultaneous screened within an actionable timeframe (Basile et al., 2018). Initially the QIAstat-Dx RP2.0 and the BioFire RP2.1 was evaluated for the detection of SARS-CoV-2 from NPS, covering a wide range of Ct values. To determine a baseline prevalence and viral load in the SARS-CoV-2 samples at the UMCG, the distribution of Ct values over a period of 17 months were initially plotted (Figure 1). Measuring Ct values are important to monitor the patient and assist infection and control, particularly as Ct values can fluctuate over time, depending on the pre-symptomatic and symptomatic phases (Rabaan et al., 2021). The high Ct values observed from UMCG patients and workers could have resulted from the increased frequency of testing and follow-up testing, regardless if the patient or worker had a milder disease, which can correspond to a higher Ct value (Fuller et al., 2013; Trunfio et al., 2021). Alternatively, it could be that we are detecting the virus earlier, before the patient or worker was particularly ill and sought medical care. Patients tested through the Public Health Service appeared to have lower Ct values which could suggest they are detecting more acute infections at the time of sampling.

The Analytical Q panel for SARS-CoV-2 from Qnostics was used to determine the LOD for the QIAstat-Dx RP2.0 and the BioFire RP2.1 (Table 1). While the BioFire RP2.1 had 100% concordance with the LDT (50 dC/mL), the QIAstat-Dx RP2.0 could only detect SARS-CoV-2 at 500 dC/mL. The fact that SARS-CoV-2 was not found by the QIAstat-Dx RP2.0 at these loads could be potentially problematic, given that this patient population has been shown to have relatively high Ct values for this target (Figure 1). This does suggest that some acute infections with higher Ct values (lower viral loads) could be missed. Differences in sensitivity between the two platforms could be due to the fact that BioFire RP2.1 is based on a nested PCR, which typically has higher sensitivity (Shaffaf and Ghafar-Zadeh, 2021), or variation in gene targets or chemistries. Nevertheless, the results achieved for the QIAstat-Dx RP2.0 have concordance with the LOD described in the instruction manual. In addition, a previous study which evaluated the GeneFinder™ COVID-19 Plus RealAmp kit using the ELITe InGenius platform found a LOD of 500 dC/mL (RdRp and E genes), which was similarly observed in the QIAstat-Dx RP2.0 (Gard et al., 2022).

A difference in clinical sensitivity in the QIAstat-Dx RP2.0 and the BioFire RP2.1 for SARS-CoV-2 was observed, with 85% and 97%, respectively. Clinical sensitivity of SARS-CoV-2 in the QIAstat-Dx RP2.0 has been investigated previously, with one study determining 100% concordance with the LDT (*n* = 17 samples), while other studies reported a PPA of 90% (*n* = 120 samples) and 94.32% (*n* = 88 samples) (Visseaux et al., 2020; Gupta et al., 2021; Lebourgeois et al., 2021). The latter study also reported a failure rate of 7.5% (*n* = 13) in the QIAstat-Dx RP2.0 (Lebourgeois et al., 2021), which was similarly observed in this study with 6.1% (2 samples). Additionally, four samples previously positive for SARS-CoV-2 in the LDT, were negative in the QIAstat-Dx RP2.0, suggesting poorer sensitivity for samples with higher Ct values. A possible explanation for the false negative results in QIAstat-Dx RP2.0 (Ct 22) and in the BioFire RP2.1 (Ct 23) could be RNA degradation, too many freeze-thaw cycles or mutations at the primer binding sites (Kanji et al., 2021; Mouliou and Gourgoulianis, 2021). Other studies investigating SARS-CoV-2 in the BioFire RP2.1 have found a PPA of 100% (*n* = 25 samples) and 98% (*n* = 49 samples) in clinical samples (Creager et al., 2020; Eckbo et al., 2021). In this small cohort, the BioFire RP2.1 did not report any instrument failures, however a previous study has reported a failure rate of 2% (*n* = 2 samples) (Creager et al., 2020).

In comparison to the BioFire RP2.1, the QIAstat-Dx RP2.0 also reports Ct values for test interpretation. This can help toward estimating the viral burden and association with disease severity. Although Ct values are generally not comparable between platforms, the Ct values in the QIAstat-Dx RP2.0 differed substantially from the LDT and were approximately five Ct values higher (Figure 2).

False positive results were not observed for SARS-CoV-2; therefore, this study can report a negative percent agreement (NPA) of 100% for both platforms. False positives can lead to unnecessary isolation for the patient and delay a true diagnosis. Previous studies investigating the QIAstat-Dx RP2.0 have reported a NPA of 90.48% (*n* = 189 samples) and 93% (*n* = 29 samples) (Visseaux et al., 2020; Lebourgeois et al., 2021), while studies investigating the BioFire RP2.1 have reported a NPA of 100% (*n* = 5 samples) (Trunfio et al., 2021), (*n* = 49 samples) (Creager et al., 2020).

Multiplex panels from Qnostics were tested on both the QIAstat-Dx RP2.0 and the BioFire RP2.1 and exhibited a similar performance (Tables 2, 3). However, in pool 1, the QIAstat-Dx RP2.0 only detected PIV1. This could be accredited to competition, as single-infections for PIV1, PIV2, and PIV4 were detected by the QIAstat-Dx RP2.0 (Table 4). As no other respiratory pathogen was detected in pools 1–4 other than what was indicated, it suggests there was no cross-reaction.

The QIAstat-Dx RP2.0 detected 113/137 targets for single infections at first attempt (Table 4). The device presented a 2.81% (*n* = 4 samples) failure rate for various reasons (IC, cartridge or test failure). The QIAstat-Dx RP2.0 performed well in most of the single infections, obtaining an overall PPA of 82% (Table 4). However, for the HRV/EV target, the lowest PPA (67%) was obtained. This could be a problem as HRV/EV is our most frequently identified target (Supplementary Figure 2). Discrepant results among these targets on the QIAstat-Dx RP2.0 and other syndromic panels have been described previously (Leber et al., 2020; Boers et al., 2021). Although some of these targets are considered a low public health risk (CE-IVD) or linked to self-limited disease (Hollestelle and de Boer, 2008), the high prevalence of positive specimens could render these viruses relevant (Supplementary Figure 2). Similar results were found among the multiple infections, with HRV/EV being the most frequently found target with discrepancies (Table 5). Although a PPA of 91.2% for the HRV/EV target has been described in the QIAstat-Dx RP2.0 manual, this was not observed in this study.

This study had some limitations. Firstly, as our data set is small for some respiratory targets, definitive conclusions are not necessarily able to be formed at this point. Secondly, as some samples did not contain enough volume, further comparisons or repeat testing, both on the QIAstat-Dx RP2.0 and BioFire RP2.1 could not always be performed. Thirdly, additional sample freezing or thawing could have impacted the results. Finally, non-SARS-CoV-2 viruses were not re-evaluated in the BioFire RP2.1, due to constraints in samples volume and time. However, according to the manufacturer, as the other respiratory targets were not altered in the update, it should not impact the results.

## Conclusion

In conclusion, this study has shown that the QIAstat-Dx RP2.0 and BioFire RP2.1 offer comparative performances; however, differ slightly in analytical and clinical sensitivity for SARS-CoV-2. A rapid and accurate differential diagnosis will ensure the most appropriate patient management decision. Point-of-care platforms are the way to move forward, however improvements are necessary such as the LOD and quantitative information.

## Data Availability Statement

The original contributions presented in the study are included in the article/Supplementary Material, further inquiries can be directed to the corresponding author.

## Author Contributions

HC: study design, laboratory and data analysis, writing—original draft preparation, writing—reviewing, and editing. MG: laboratory and data analysis, writing—reviewing, and editing. EL-F: study design, laboratory and data analysis, writing—reviewing, and editing. HN and LG: conceptualization, editing, and supervision. All authors contributed to the article and approved the submitted version.

## Conflict of Interest

Qiagen provided the QIAstat-Dx respiratory SARS-CoV-2 panel V2 test cartridges used in this study but did not take part on the study design, data collection, analysis, or manuscript preparation. The authors declare that the research was conducted in the absence of any commercial or financial relationships that could be construed as a potential conflict of interest.

## Publisher’s Note

All claims expressed in this article are solely those of the authors and do not necessarily represent those of their affiliated organizations, or those of the publisher, the editors and the reviewers. Any product that may be evaluated in this article, or claim that may be made by its manufacturer, is not guaranteed or endorsed by the publisher.
