# Supplementary material for: Evaluation of the QIAstat-Dx RP2.0 and the BioFire FilmArray RP2.1 for the Rapid Detection of Respiratory Pathogens Including SARS-CoV-2
Source: Front Microbiol. 2022 Mar 24;13:854209. doi: 10.3389/fmicb.2022.854209 (PMC8989387; doi:10.3389/fmicb.2022.854209)
Supplement: Supplementary file 1 [file Data_Sheet_1.docx]

Supplementary Material

# Supplementary Methods and Methods

## Laboratory developed test (human rhinovirus/enterovirus)

To differentiate between a rhinovirus and enterovirus following the BioFire RP2.0 or laboratory developed respiratory screen, a RT-qPCR targeting the 5’NTR region was performed. Briefly, nucleic acids were extracted from 190μL of sample using the NucliSense EasyMag (BioMérieux) and eluted in 110µL. PDV severed as an IC. A multiplex RT-qPCR using the Fast Virus 1-Step kit (ThermoFisher Scientific, Waltham, USA) was performed using 10μL of extracted RNA in a total reaction volume of 25μL (1). Amplification was performed on an ABI7500 (Life Technologies, Carlsbad, USA) with the following PCR cycling conditions: 2min 50°C, 20s 95°C, followed by 45 cycles of 3s 95°C and 32s at 60°C.

## Evaluating dual sensitivity

The clinical sensitivity of dual infections on the QIAstat-Dx RP2.0 was evaluated by creating artificial co-infections from common respiratory pathogens within a single sample and determining the limit of detection. The artificial co-infections were created to increase the number of targets tested per cartridge. The following viral targets: SARS-CoV-2, influenza A virus H3, rhinovirus, parainfluenza virus 1 and coronavirus OC43 were preselected from patient samples collected between 2016 and 2020. Each sample containing the selected viral target had been previously tested on the LDT respiratory panel to determine the Ct value. Initially, samples containing the target respiratory pathogen were diluted to a Ct value of 24 and then combined together to create three separate panels. Panel 1 contained SARS-CoV-2 and influenza A virus H3, panel 2 contained SARS-CoV-2 and rhinovirus and finally panel 3 contained parainfluenza virus 1 and coronavirus OC43. A half log dilution series (10^2^ to 10^6^) was then performed to determine the LOD. Each artificial panel was run in duplicate on the QIAstat-Dx (RP2.0).

## References

Poelman R, Schölvinck EH, Borger R, Niesters HG, van Leer-Buter C. The emergence of enterovirus D68 in a Dutch University Medical Center and the necessity for routinely screening for respiratory viruses. J Clin Virol. 2015;62:1-5. doi:10.1016/j.jcv.2014.11.011

# Supplementary Figures and Tables

## Tables

## Table S1: Demographics of patients with detected single respiratory pathogens

| **Respiratory target from**  **BioFire (RP2.0)** | **Total number included** | **Gender** | | **Age range (years)** | | |
| --- | --- | --- | --- | --- | --- | --- |
|  |  | **Male** | **Female** | **<6** | **6-18** | **>18** |
| Adenovirus | 1 | 0 | 1 | 0 | 0 | 1 |
| Bocavirus | 0 | 0 | 0 | 0 | 0 | 0 |
| Coronavirus 229E | 1 | 0 | 1 | 0 | 0 | 1 |
| Coronavirus HKU1 | 3 | 0 | 3 | 0 | 0 | 3 |
| Coronavirus NL63 | 6 | 4 | 2 | 0 | 0 | 6 |
| Coronavirus OC43 | 5 | 2 | 3 | 0 | 0 | 5 |
| Human Metapneumovirus A/B | 9 | 5 | 4 | 0 | 0 | 9 |
| Human Rhinovirus/Enterovirus | 27 | 11 | 16 | 0 | 1 | 26 |
| Influenza A | 2 | 1 | 1 | 0 | 0 | 2 |
| Influenza A subtype H1 | 0 | 0 | 0 | 0 | 0 | 0 |
| Influenza A subtype H1N1/2009 | 8 | 4 | 4 | 0 | 0 | 8 |
| Influenza A subtype H3 | 16 | 9 | 7 | 1 | 0 | 15 |
| Influenza B | 0 | 0 | 0 | 0 | 0 | 0 |
| Parainfluenza virus 1 | 8 | 3 | 5 | 0 | 0 | 8 |
| Parainfluenza virus 2 | 1 | 0 | 1 | 0 | 0 | 1 |
| Parainfluenza virus 3 | 0 | 0 | 0 | 0 | 0 | 0 |
| Parainfluenza virus 4 | 3 | 2 | 1 | 0 | 0 | 3 |
| Respiratory Syncytial virus A/B | 11 | 7 | 4 | 0 | 0 | 11 |
| SARS-CoV-2 | 33 | 22 | 11 | 0 | 0 | 33 |
| *Bordetella pertussis* | 0 | 0 | 0 | 0 | 0 | 0 |
| *Legionella pneumophila* | 0 | 0 | 0 | 0 | 0 | 0 |
| *Mycoplasma pneumoniae* | 3 | 1 | 2 | 0 | 0 | 3 |
| Total | 137 | 71 | 66 | 1 | 1 | 135 |

## Table S2: Demographics of patients with detected multi-respiratory pathogens

| **Sample number** | **Respiratory targets from**  **BioFire (RP2.0)** | **Gender** | | **Age range (years)** | | |
| --- | --- | --- | --- | --- | --- | --- |
|  |  | **Male** | **Female** | **<6** | **6-18** | **>18** |
| 1 | HRV/EV and PIV1 | 1 | 0 | 0 | 0 | 1 |
| 2 | HRV/EV and PIV4 | 1 | 0 | 0 | 0 | 1 |
| 3 | HRV/EV and RSV | 1 | 0 | 0 | 0 | 1 |
| 4 | HRV/EV and INFH3 | 1 | 0 | 0 | 0 | 1 |
| 5 | HRV/EV and PIV4 | 1 | 0 | 0 | 0 | 1 |
| 6 | HRV/EV and CoV-OC43 | 0 | 1 | 0 | 0 | 1 |
| 7 | HRV/EV, AV and RSV | 0 | 1 | 1 | 0 | 0 |
| 8 | HRV/EV and RSV | 0 | 1 | 0 | 0 | 1 |
| 9 | HRV/EV and INFH3 | 1 | 0 | 0 | 0 | 1 |
| 10 | HRV/EV and BP | 1 | 0 | 0 | 0 | 1 |
| 11 | HRV/EV, AV and INFH3 | 1 | 0 | 1 | 0 | 0 |
| 12 | HRV/EV and INFH3 | 0 | 1 | 0 | 0 | 1 |
| 13 | CoV-OC43 and RSV | 0 | 1 | 0 | 0 | 1 |
| 14 | CoV-OC43, INFH3, CoV-HKU1 | 0 | 1 | 0 | 0 | 1 |
| 15 | CoV-NL63 and INFAH3 | 0 | 1 | 0 | 0 | 1 |
| 16 | CoV-NL63 and INFAH3 | 0 | 1 | 0 | 0 | 1 |
| Total | | 8 | 8 | 2 | 0 | 14 |

Abbreviations: Human rhinovirus/enterovirus; HRV/EV, Adenovirus; AV, Bordetella Virus Type pertussis; BP, PIV1; Human Parainfluenza Virus Type 1, PIV4; Human Parainfluenza 4, RSV; Respiratory syncytial virus, INFH3; Influenza A subtype H3, CoV-OC43; coronavirus OC43, AV; adenovirus, HKU1; Coronavirus HKU1, CoV-NL63; coronavirus NL63, Ct; cycle threshold.

## Table S3: Primers and probes for the laboratory developed respiratory screen

| **Target** | **Oligo name** | **Sequence (5′ → 3′)** | **Primer Concentration** |
| --- | --- | --- | --- |
| Coronavirus 229E | 229E-fwd-TM | cgcaagaattcagaaccagag | 10pmol/ul |
|  | 229E-PB-Cy5 | ccacacttcaatcaaaagctcccaaatg | 5pmol/ul |
|  | 229E-rev-TM | gggagtcaggttcttcaacaa | 15pmol/ul |
| Adenovirus | Adenoquant1 | gccacggtggggtttctaaactt | 10pmol/ul |
|  | Adenoquant2 | gccccagtggtcttacatgcacatc | 45pmol/ul |
|  | Adenoquant-probe | tgcaccagacccgggctcaggtactccga | 5pmol/ul |
| Bocavirus | Boca-TM-fwd | gcccgatccgacacagtg | 45pmol/ul |
|  | Boca-TM-probe | agagaggctcgggctcatatcatcaggaa | 5pmol/ul |
|  | Boca-TM-rev | tgcaagacgataggtggctg | 45 pmol/ul |
| Coronavirus HKU1 | HKU1_p | cgcctggtacgattttgcctcaaggct | 5pmol/ul |
|  | HKU1_rv | ttagaagcagaccttcctgagcc | 15pmol/ul |
|  | HKU1-fw | cacttctaytccctccgatgtttc | 15pmol/ul |
| Human metapneumovirus A/B | hMPV_P | tgcaatgatgarggtgtcactgcngttg | 10pmol/ul |
|  | hmpv-fwd-TM | catataagcatgctatattaaaagagtctc | 30pmol/ul |
|  | hmpv-rev-TM | cctatttctgcagcatatttgtaatcag | 30pmol/ul |
| Influenza A | infa_fwd | cttctraccgaggtcgaaacgta | 45pmol/ul |
|  | infa_probe1 | tcaggccccctcaaagccgaga | 5pmol/ul |
|  | infa_probe2 | tcaggccccctcaaagccgaaa | 5pmol/ul |
|  | infa_rev | tcttgtctttagccaytccatgag | 45pmol/ul |
| Influenza B | infb-NSfwdB | gracaacatgaccacaacacaaat | 30pmol/ul |
|  | infb-NSprobeB | cgggagcaaccaatgccaccataaa | 5pmol/ul |
|  | infb-NSrevB | cactccaraattcctgcttcaaa | 45pmol/ul |
| Coronavirus NL63 | NL63-fwd | gaa gcg tgt tcc tac cag aga | 30pmol/ul |
|  | NL63-PB-Cy5 | aaatgttattcagtgctttggtcctcgtga | 10pmol/ul |
|  | NL63-rev | gaatcccccatattgtgattaaa | 45pmol/ul |
| Coronavirus OC43 | OC43TM_fwd1 | cgatgaggctattccgactaggt | 30pmol/ul |
|  | OC43TM_prb1 | tccgcctggcacggtactccct | 5pmol/ul |
|  | OC43TM_rev1 | ccttcctgagccttcaatatagtaacc | 45pmol/ul |
| Parainfluenza virus 1 | PARA-1-FWD-TM | tgatttaaacccggtaatttctcat | 15pmol/ul |
|  | PARA-1-probe-TM | acgacaacaggaaatc | 5pmol/ul |
|  | PARA-1-REV-TM | ccttgttcctgcagctattacaga | 15pmol/ul |
| Parainfluenza virus 3 | PARA-3-FWD-TM | ggaccagggatatactayaaa | 45pmol/ul |
|  | PARA-3-probe-TM | atctgyaacacaactggrtgtccygggaa | 5pmol/ul |
|  | PARA-3-REV-TM | ttgaccatcctyctrtctgaa | 45pmol/ul |
| Parainfluenza virus 4 | PARA-4-FWD-TM | atggtgggagayattgcaaa | 30pmol/ul |
|  | PARA-4-probe-TM | atatagcyaatgtcggaatgagygcgttcttt | 10pmol/ul |
|  | PARA-4-REV-TM | ccaagccgaacttaagygtaa | 30pmol/ul |
| phocine distemper virus^*1^ | PDV fwd | cgggtgccttttacaagaac | 30pmol/ul |
|  | PDV rev | ttctttcctcaacctcgtcc | 40pmol/ul |
|  | PDV_MGB_NED | aag ggc caa ttc t | 5pmol/ul |
| Parainfluenza virus 2 | Piv2HNfwdA | ctgcatcgctyttttacaggatc | 30pmol/ul |
|  | piv2HNrevA | cttgttgcattrcatggcat | 30pmol/ul |
|  | piv2probeA | tcattgaggctcaatgggtaccgtcctatc | 10pmol/ul |
| Human rhinovirus/Enterovirus | Rhino-lna-pr2 | yg+gg+ay+gggaccaact | 12,5pmol/ul |
|  | HRVHEVfw | cggcccctgaatgygg | 37,5pmol/ul |
|  | Rhi-asense | tggaaacacggacacccaa | 37,5pmol/ul |
|  | HEV1-lna-pr4 | yg+tg+gcggaaccga | 6,25pmol/ul |
|  | HEV2-lna-pr2 | yg+ca+gc+ggaaccgact | 6,25pmol/ul |
| Respiratory Syncytial virus A | RSV-A-R-fw | ctcaatttcctcacttctccagtgt | 25pmol/ul |
|  | RSV-A-R-probe | tcccattatgcctaggccagcagca | 10pmol/ul |
|  | RSV-A-R-rv | cttgattcctcggtgtacctctgt | 25pmol/ul |
| Respiratory Syncytial virus B | RSV-B-R-drfly | tcccattatgcctagacctgctgcattg | 10pmol/ul |
|  | RSV-B-R-fw | ttcctaacttctcaagtgtggtccta | 15pmol/ul |
|  | RSV-B-R-rv | ctggtttcttggcgtacctctatac | 15pmol/ul |
| SARS-CoV-2^*2^ | E_Sarbeco_F | acaggtacgttaatagttaatagcgt | 20pmol/ul |
|  | E_Sarbeco_P1 | acactagccatccttactgcgcttcg | 10pmol/ul |
|  | E_Sarbeco_R | atattgcagcagtacgcacaca | 20 pmol/ul |

Abbreviation: SARS-CoV-2; severe acute respiratory syndrome coronavirus 2.^*1^ internal control ^*2^ target added to respiratory screen after completion of the study.

## Table S4: BioFire RP2.1 and QIAstat-Dx 2.0 targets

| **Panel** | **BioFire RP2.1** | **QIAstat-Dx 2.0** |
| --- | --- | --- |
| **Viruses** | Adenovirus | Adenovirus |
|  | N/A | Bocavirus |
|  | Coronavirus 229E | Coronavirus 229E |
|  | Coronavirus HKU1 | Coronavirus HKU1 |
|  | Coronavirus NL63 | Coronavirus NL63 |
|  | Coronavirus OC43 | Coronavirus OC43 |
|  | Human metapneumovirus A/B | Human metapneumovirus A/B |
|  | Human rhinovirus/Enterovirus | Human rhinovirus/Enterovirus |
|  | Influenza A | Influenza A |
|  | Influenza A subtype H1 | Influenza A subtype H1 |
|  | Influenza A subtype H1N1/2009 | Influenza A subtype H1N1/2009 |
|  | Influenza A subtype H3 | Influenza A subtype H3 |
|  | Influenza B | Influenza B |
|  | Parainfluenza virus 1 | Parainfluenza virus 1 |
|  | Parainfluenza virus 2 | Parainfluenza virus 2 |
|  | Parainfluenza virus 3 | Parainfluenza virus 3 |
|  | Parainfluenza virus 4 | Parainfluenza virus 4 |
|  | Respiratory Syncytial Virus A/B | Respiratory Syncytial virus A/B |
|  | SARS-CoV-2^*1^ | SARS-CoV-2 |
| **Bacteria** | *Bordetella parapertussis* | N/A |
|  | *Bordetella pertussis* | *Bordetella pertussis* |
|  | *Chlamydia pneumoniae* | N/A |
|  | *Mycoplasma pneumoniae* | *Mycoplasma pneumoniae* |
|  | N/A | *Legionella pneumophila* |

^*1^ Not present in the BioFire respiratory panel, N/A; not applicable

## Figures

| a) Clinical sensitivity panel 1  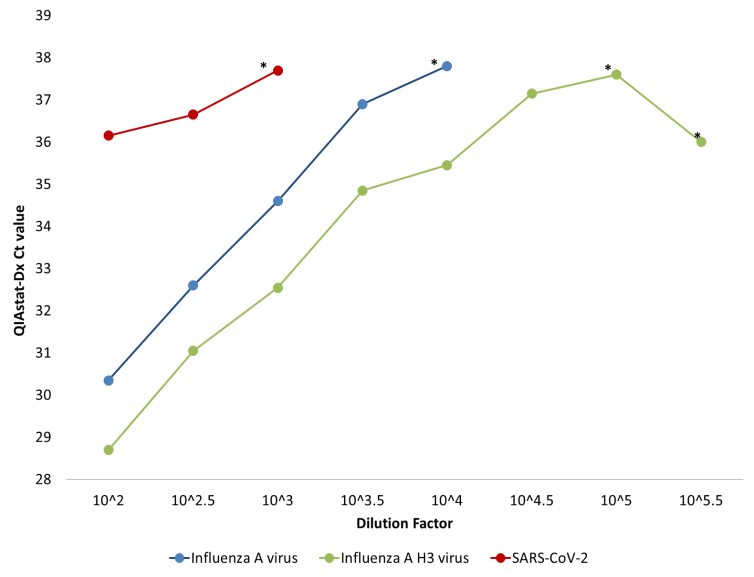 | b) Clinical sensitivity panel 2  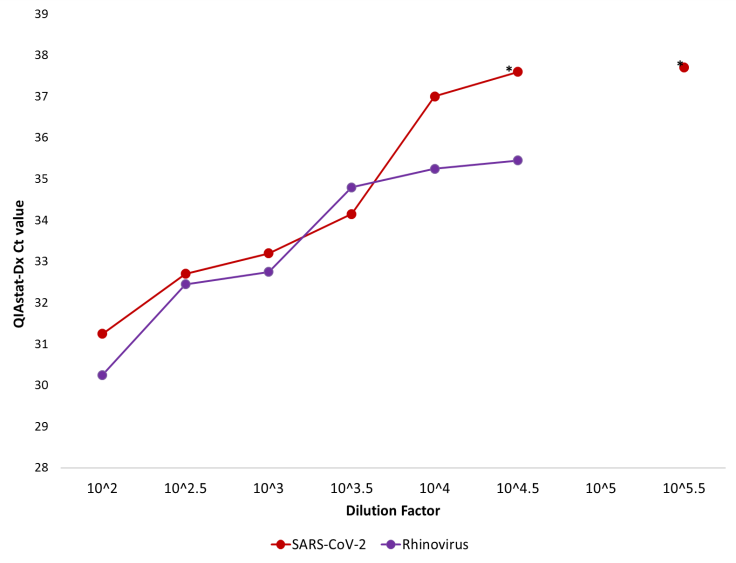 |
| --- | --- |
| c) Clinical sensitivity panel 3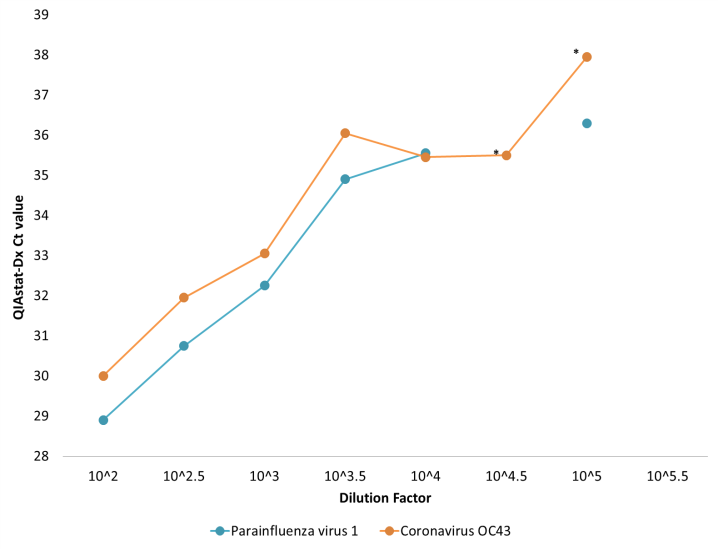 |  |
| **Supplementary Figure 1:** Clinical sensitivity of selected viral targets from patient samples. a) Clinical sensitivity panel 1.b) Clinical sensitivity panel 2. c) Clinical sensitivity panel 3. Overall, the majority of viral targets had a LOD of Ct 37 (Ct 35.4-38.1) on the QIAstat-Dx (RP2.0). However, detection was not always linear as influenza A virus H3 in panel 1 had a stronger viral load at log10^5.5^, compared to log10^5^. Similarly, SARS-CoV-2 in panel 2, along with parainfluenza virus 1 and CoV-OC43 in panel 3, had a negative result prior to becoming detectable again. CoV-OC43 had the highest LOD with a Ct value of 38.1; meanwhile, rhinovirus had the lowest LOD with a Ct value of 35.**^*^**Only single detection (not in duplicate) | |


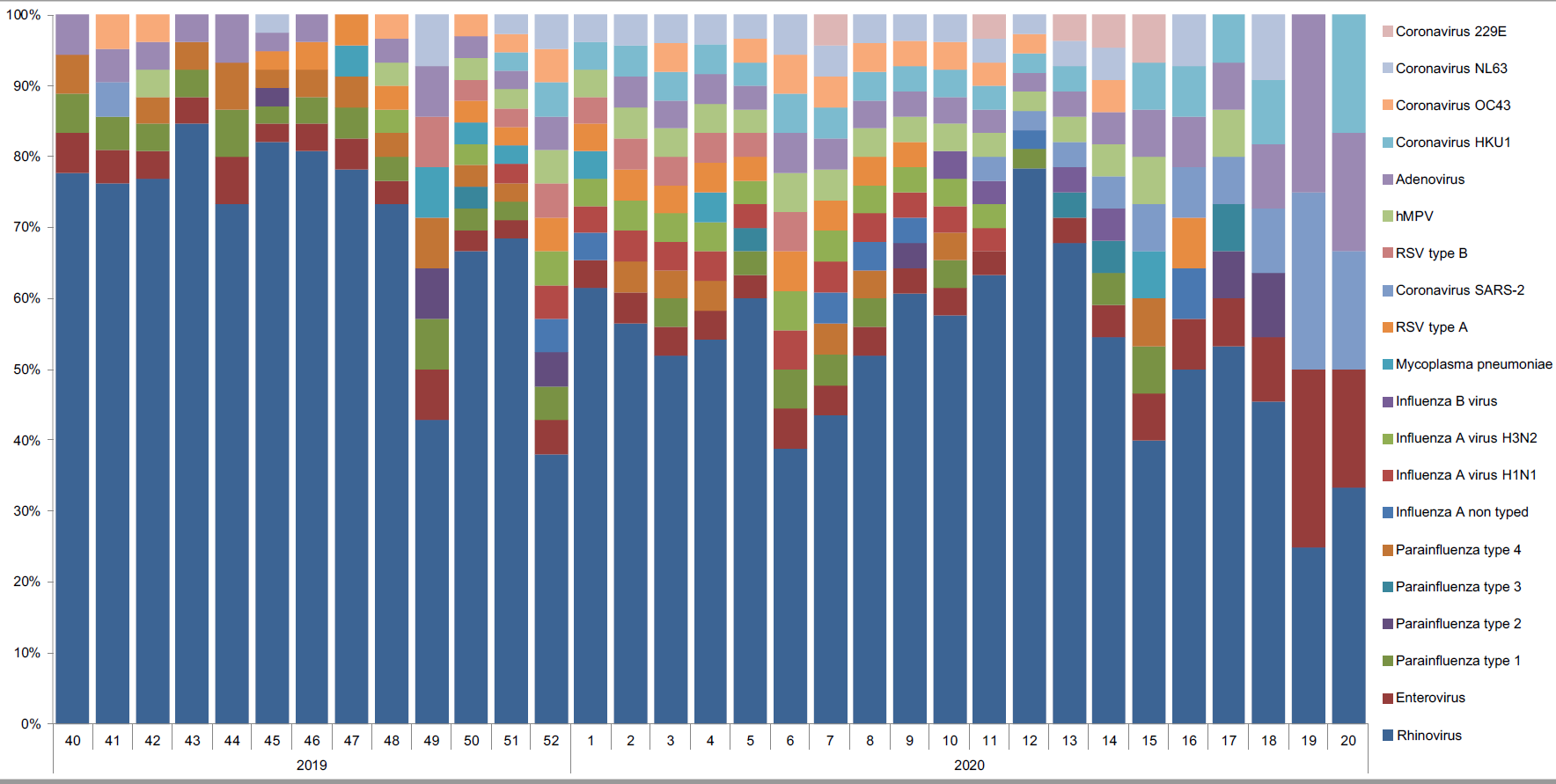


**Supplementary Figure 2.** The percentage of positive specimens at the UMCG during the 2019/2020 respiratory season week 40 of 2019 through to week 20 of 2020. Displayed by week of sampling. Abbreviations: hMPV; Human metapneumovirus, RSV; Respiratory Syncytial Virus
